# The Ocular Microbiome in Stevens-Johnson Syndrome

**DOI:** 10.3389/fmed.2021.645053

**Published:** 2021-05-07

**Authors:** Thanachaporn Kittipibul, Vilavun Puangsricharern

**Affiliations:** ^1^Excellence Center for Cornea and Limbal Stem Cell Transplantation, Department of Ophthalmology, Faculty of Medicine, King Chulalongkorn Memorial Hospital, Chulalongkorn University, Bangkok, Thailand; ^2^Department of Ophthalmology, Faculty of Medicine, Chulalongkorn University, Bangkok, Thailand

**Keywords:** Stevens-Johnson syndrome, ocular surface, ocular surface disease, microbiome, microbial community

## Abstract

The ocular surface microbiome is an essential factor that maintains ocular surface homeostasis. Since the ocular surface is continuously exposed to the external environment, its microbiome, tears, and local immunity are vital for maintaining normal conditions. Additionally, this microbiome helps prevent pathogen colonization, which commonly leads to opportunistic infection. The abnormal ocular surface microbiome has previously been reported in several conditions, including dry eyes, allergy, blepharitis, graft-versus-host disease (GVHD), and Stevens-Johnson syndrome (SJS). Several approaches were applied to identify the ocular microbiome, including conventional culture techniques and molecular sequencing techniques. By using 16s rRNA sequencing, alterations in the type, proportion, and composition of bacterial communities, described by alpha (α)-and beta (β)-diversity, were observed in SJS patients compared to the healthy group. Conventional culture techniques indicated a higher number of positive bacterial cultures in the SJS group, with a predominance of gram-positive cocci and gram-positive bacilli. Besides, there are increased variations and multiple detections of bacterial genera. Taken together, SJS causes structural changes in the ocular surface and significantly affects its microbiome. Further studies into the area of temporal relationship, metagenomics, proteomics, and metabolomics analysis of the microbiome will lead to a better understanding of this disease. Finally, the treatment using prebiotics and probiotics to re-establish the normal ocular ecosystem and bring back a healthy ocular surface await confirmation.

## Introduction

The ocular surface appears to be the most anterior part of the eye, exposed to the external environment. Several barriers, such as tear film, local immunity, and commensal bacteria, play essential roles on the ocular surface to protect and maintain homeostasis. In a healthy ocular surface, the microbiome is an essential factor that maintains ocular surface homeostasis and strengthens innate immunity by increasing immune effectors, particularly IgA, in tear films (1).

Microbial communities in human bodies are commonly specific in each body site. Differences in the microbial composition are associated with health and disease. In healthy subjects, a predominance of Bacteroidetes was observed in the gastrointestinal system, while Actinobacteria was a major phylum isolated from the skin. Besides, alteration of microbial communities may be related to the pathogenesis of some diseases. *Lactobacillus*, which commonly colonizes and stabilizes pH in the vagina, is shifting in bacterial vaginosis. Thus, the relationship between microbial communities and human bodies is another essential factor that regulates health status (2, 3).

Previous studies have shown a low bacterial load on a healthy ocular surface. There were 10–13% of positive cultures (4, 5) with a predominance of coagulase-negative *staphylococci, Corynebacterium*, and *Propionibacterium* (6, 7). Although conventional culture techniques can detect bacteria, the technique is limited for conditions such as slow-growing and unculturable microorganisms. Genome sequencing was recently applied for microbial detection, which showed a higher sensitivity by reporting several prominent bacterial genera, including *Propionibacterium, Corynebacteria, Staphylococcus*, and *Streptococcus* in healthy subjects (8, 9). Furthermore, Doan et al. identified 42 genera from conjunctival samples of healthy with highest confidence genera of *Corynebacteria, Propionibacteria*, and *Staphylococcus* (10).

Several conditions, such as contact lens, topical medications, dry eyes, meibomian gland dysfunction (MGD), allergy, trachoma, blepharitis, graft-versus-host disease (GVHD), and Stevens-Johnson syndrome (SJS), can disrupt the ocular surface microbiome. Shin et al. studied the impact of the contact lens on the ocular microbiome. They reported an increased proportion of *Methylobacterium, Lactobacillus, Acinetobacter*, and *Pseudomonas* in lens wearers compared with non-wearers (11). This study also reported a more remarkable similarity in the ocular microbiome and skin microbiome in contact lens wearers (11). Further, Graham et al. reported altering the ocular microbiome in patients with dry eyes, detecting atypical bacterial genera such as *Rhodococcus, Klebsiella*, and *Erwinia* (12). The shifting of colonized genera was also reported in MGD with an increased number of potentially pathogenic strains, including *Staphylococcus epidermidis, Propionibacterium acne, Coryneform bacteria*, and *Staphylococcus aureus* (13). Similar trend of shifting colonization was also identified in GVHD patients. Higher ratio of culture-positive and multiple microbial detection were observed in GVHD compared to non-GVHD. In addition, *Alpha-haemolytic Streptococcus, Haemophilus influenza, and Enterobacter cloacae*, which were defined as pathogenic species, were mostly isolated from GVHD (14).

## Stevens-Johnson Syndrome as a Severe Ocular Surface Disorder

SJS is a rare but severe disorder that affects the skin, mucous membrane, genitals, and eyes. The etiology is immune-mediated and can be triggered mostly by drugs, followed by viral or Mycoplasma infections. About 20–79% of survivors will experience severe ocular complications from chronic inflammation, desiccation, and scarring leading to blindness (15, 16).

Ocular infection occurs quite common in chronic SJS, most of which is severe, recurrent, and challenging to treat due to the occurrence of multidrug-resistant organisms. Nouri et al. reported incidences of endophthalmitis after keratoprosthesis transplantation in 13 of 108 patients. SJS was identified as a significant cause of endophthalmitis, which accounted for 39% of this report (17). Gunasekaran et al. reported ocular surface flora in chronic limbal stem cell deficiency and found culture-positive in all 13 specimens. Ten of thirteen cases were diagnosed as SJS, which were positive for *Staphylococcus epidermidis* (6 cases), *Staphylococcus aureus* (3 cases), and multiple microorganisms (1 case) (18). Furthermore, SJS was probably associated with multiple drug-resistant microorganisms. Sotozono et al. found 6 of 10 cases of methicillin-resistant *Staphylococcus aureus* (MRSA) keratitis in SJS patients (19). These findings, taken together, imply that ocular infection in SJS may be associated with abnormal microbial colonization on the ocular surface.

## Role Of the Ocular Surface Microbiome in SJS

The normal ocular microbiota helps preventing colonization by pathogenic species, which associated with various pro-inflammatory states. The analysis of the ocular microbiome composition is crucial for understanding the pathophysiology of various ophthalmic diseases. The ocular surface innate immune system recognizes and responds to this microbiota through different pathways, including the “Pattern Recognition Receptors” (PRRs) through Toll-Like Receptors (TLRs), which are the transmembrane receptors expressed on different cells (20). Under normal state, the ocular surface immune system maintains the “immune silence” condition when several unique mechanisms prevent the unnecessary inflammatory response to the normal bacterial flora (21).

In SJS, the viral infection, genetic factors, or other environmental factors trigger the innate immune system via the TLR pathway, leading to the expression of different pro-inflammatory cytokines, chemokines, or other molecules as proposed by Ueta et al. (22). These conditions lead to inflammation that can compromise visual function in patients. The same authors also proposed that abnormal host mucosal immunity will affect the normal polyclonality of the commensal bacteria, resulting in the monoclonality of bacteria that can become pathogenic (23).

Thus, the ocular surface problem in SJS potentially causes microbiome changes in terms of the bacterial load, characteristics of prominent microorganisms, the proportion of each genus or species, and diversity of microbial communities, which can lead to a greater risk of infections and chronic inflammation. Furthermore, the ocular surface microbiome may be another indicator of the severity of ocular surface diseases.

## Studies On the Ocular Microbiome in SJS Patients

Due to the rarity of SJS, there is still little information regarding the characteristics of the ocular surface microbiome and its impact on ocular SJS. Previous studies mostly demonstrated the microbial community with conventional culture techniques. According to culture condition and duration, which affect each bacteria's growth differently, these techniques detected only a fraction of the microbial community (7, 24). The 16s rRNA assays and newer genomic sequencing techniques were introduced for microbiome analysis to improve microbial detection effectiveness. This novel analyses demonstrated efficient identification of microbiota, especially in uncultivable and lower abundance genera (25).

Recently, the new 16s rRNA sequencing, named Next-Generation Sequencing (NGS) technique gave unbiased results together with high-throughput data analysis, and performed massively parallel sequencing in a shorter time (26).

We aimed to review the ocular microbiome in SJS patients and compared them with healthy subjects. We referred to both conventional culture techniques and molecular sequencing techniques to get comprehensive data.

### Conventional Culture Techniques

In our recent study (5), we collected specimens by swabbing conjunctiva and placing them in transport media. After dissolving specimens, we devided each specimens into 2 parts. The first part was placed on chocolate agar for bacterial isolation. Bacterial identification was performed by biochemical tests and the API system. We found significantly higher culture-positive rates in the SJS group. While 60% (12 of 20 eyes) of the SJS patients were culture-positive, only 10% (2 of 20 eyes) of the healthy individuals were culture-positive. Besides, multiple bacterial detections were reported only in the SJS group, similar to a previous report (4). Venugopal et al. (4) found that the most common isolates in the SJS group were *Corynebacterium spp*. (4 of 12 eyes) and *Streptococcus spp*. (4 of 12 eyes) followed by *Staphylococcus spp*. (3 of 12 eyes). Compared to healthy groups, gram-positive species are still predominant on the ocular surface. Frizon et al. also reported the microbial detection in SJS with the highest number of *Staphylococcus spp.*, followed by *Corynebacterium spp*. These results showed that gram-positive are still predominant species in SJS patients with a higher rate of culture-positive (27). Our study's conventional culture results also discovered atypical bacteria in SJS, including *Streptococcus agalactiae* and *Proteus mirabilis* (5). Frizon et al. (27) found several atypical species colonizing on the ocular surface of SJS, such as *Serratia non-liquefaciens, Escherichia coli*, and *Proteus mirabilis, Haemophilus spp*. However, there is not only an alteration of microbial colonization but also a change in the antimicrobial susceptibility. Venugopal et al. (4) reported antibiotic resistance to second-generation fluoroquinolones, similar to Frizon et al. (27) who reported antibiotic resistance to chloramphenicol and second-generation fluoroquinolones. Conversely, such resistant strains were not found in our study.

In summation, SJS, which affects anatomical structures and immune modulation (28), also commonly disrupted ocular surface homeostasis and caused dysbiosis. This condition may result in the amplification of several bacteria, which increases the possibility of bacteria detection by conventional cultures.

### Molecular Sequencing Techniques

Conventional culture techniques may not demonstrate the overall composition of the microbial community. Dong et al. observed a disparity between microbiologic and molecular approaches. Molecular methods by 16s rRNA sequencing demonstrated 59 distinct bacterial genera, which was more than three times higher diversity than culture methods. They concluded that these newer approaches might provide better pieces of knowledge (7).

In our study (5), residual specimens from conventional cultures were extracted for 16s rRNA amplification with V3-V4 regions targeting. After genome products were prepared, analysis of sequences was performed by Illumina miseq platform.

We reported SJS's core microbiome, identified as 100% of core OTUs samples matching, consisted of *Pseudoalteromonadaceae, Vibrionaceae, Burkholderiaceae, Enterobateriaceae* (5). Microbiome diversity was demonstrated by α- diversity, which showed a higher number of bacterial species (represented by observed operational taxonomic units or OTUs) in SJS (Supplementary Figure 1). Similarly, the Shannon index also demonstrated a higher species abundance in the SJS group (Supplementary Figure 2). These findings indicate a more significant variation in the microorganisms existing on the ocular surface of SJS patients. However, Zilliox et al. reported the ocular microbiome of SJS in different ways. They found that 7 SJS patients had lower diversity compared to healthy (29). The disparity among studies may be from different baseline characteristics, including geographic distribution, lifestyle, antimicrobial administration pattern, etc. Regarding geographic distribution, Deng et al. reported a difference in ocular surface microbiome from Guangzhou, Beijing, and Wenzhou. Predominance of *Propionibacterium acne* was observed in Guangzhou's subjects, while *Pseudomonas aeruginosa* was predominantly seen in Beijing's subjects. Besides, the diversities of the conjunctival microbiome were different among the three districts (30).

**Figure 1:**
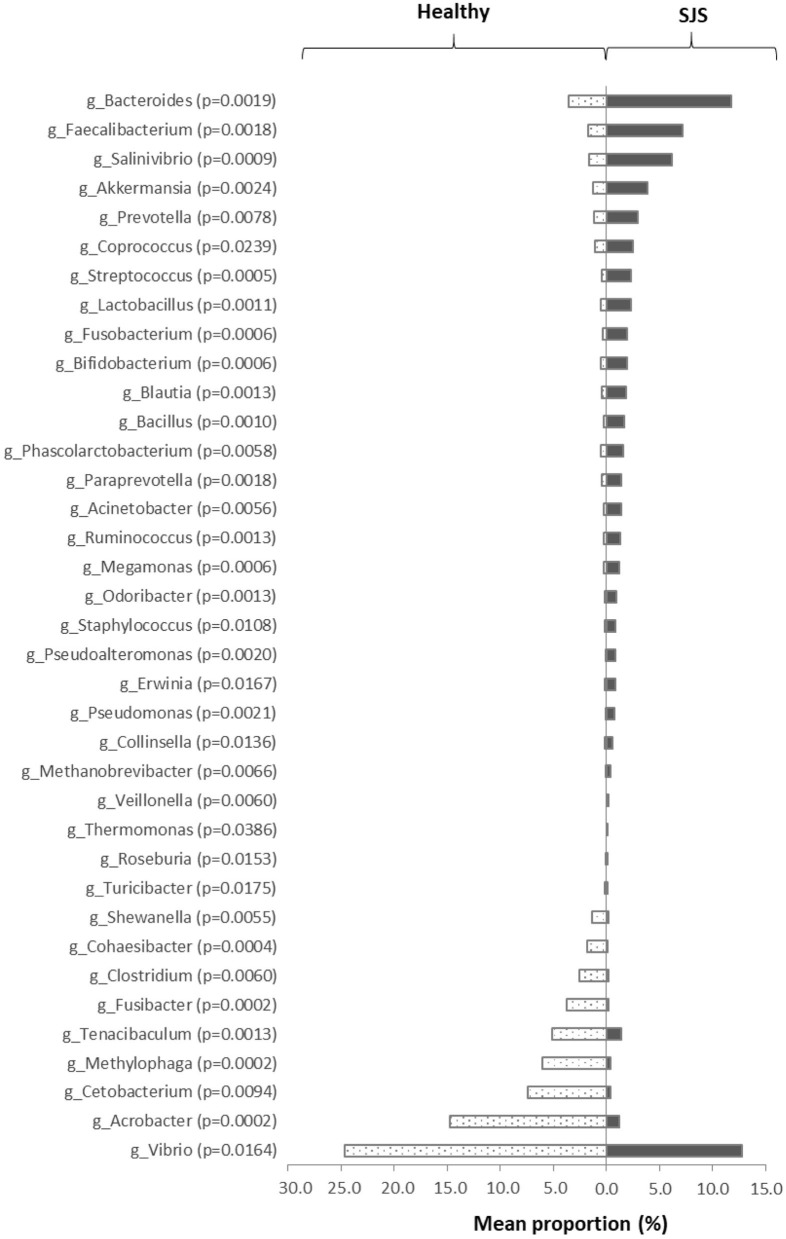
The significant difference in taxa abundance. This figure represents the significant difference in each genus' taxonomic level compared between SJS patients and healthy subjects. We analyzed the proportion of each genus by Mann–Whitney *U*-test. The *p* < 0.05 was defined as statistically significant difference.

We also compared each specific genera between SJS and healthy. There was a higher relative proportion of *Lactobacillus, Bacteroides, Pseudomonas, Staphylococcus, Streptococcus, Bacillus*, and *Acinetobacter* in the SJS group (Figure 1). Similarly, Zillox et al. reported a higher proportion of *Staphylococcus* in their study. Furthermore, our study showed an increased number of potentially pathogenic bacteria, including *Pseudomonas spp*. and *Acinetobacter spp*. in the SJS group, which may increase the risk of opportunistic infections.

Regarding the severity of SJS, we analyzed the correlation between the severity of the disease and culture-positive specimens. We found a significantly higher severity score in the culture-positive group (*p* = 0.016) (5). However, we could not demonstrate a correlation between the number of OTUs and the severity grading (Supplementary Figure 3). Further study in a larger group may illustrate the definite correlation between microbiome and disease severity.

## Future Research and Applications

Current knowledge of ocular microbiome change in SJS has mostly been uncovered. However, it remained unclear whether the microbiome's change plays a role in increasing inflammation in SJS or the disease alters the microbiome. Further studies trying to assess the temporal relationship between the two is undoubtedly warranted. Further assessments, including metagenomic profiling, protein expression, and metabolic activity, may bring us to a better perception of this disease's microbiome (31). Future studies should also be directed to identify other factors' roles in this change, such as aging, systemic diseases, surgical interventions, and antimicrobial treatment. Finally, various treatments aiming to restore the healthy ocular ecosystem should also be explored. Although probiotics and prebiotics are still not directly applied in ocular treatment, a report shows the improvement of dry eye after initiating oral prebiotic supplement (32). This new treatment may pave the way for better control of this devastating ocular surface disease.

## Author Contributions

TK and VP contributed to the writing of this review and have read and approved the final version.

## Conflict of Interest

The authors declare that the research was conducted in the absence of any commercial or financial relationships that could be construed as a potential conflict of interest. The handling Editor declared a past collaboration with one of the authors VP.
