# Supplementary figures and images for: The Ocular Microbiome in Stevens-Johnson Syndrome
Source: Front Med (Lausanne). 2021 May 7;8:645053. doi: 10.3389/fmed.2021.645053 (PMC8138458; doi:10.3389/fmed.2021.645053)

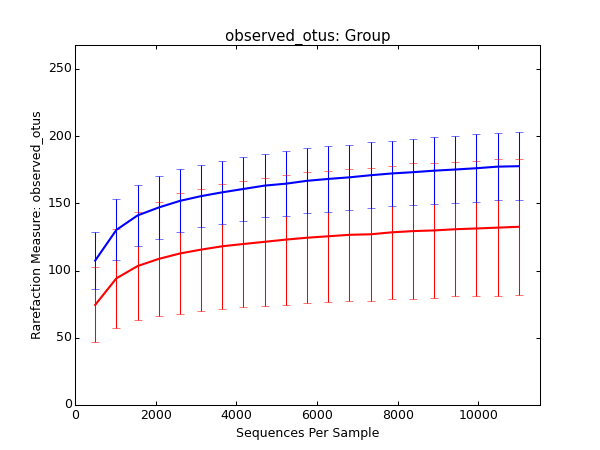

Supplement: Supplementary file 1 [file Image_1.JPEG]

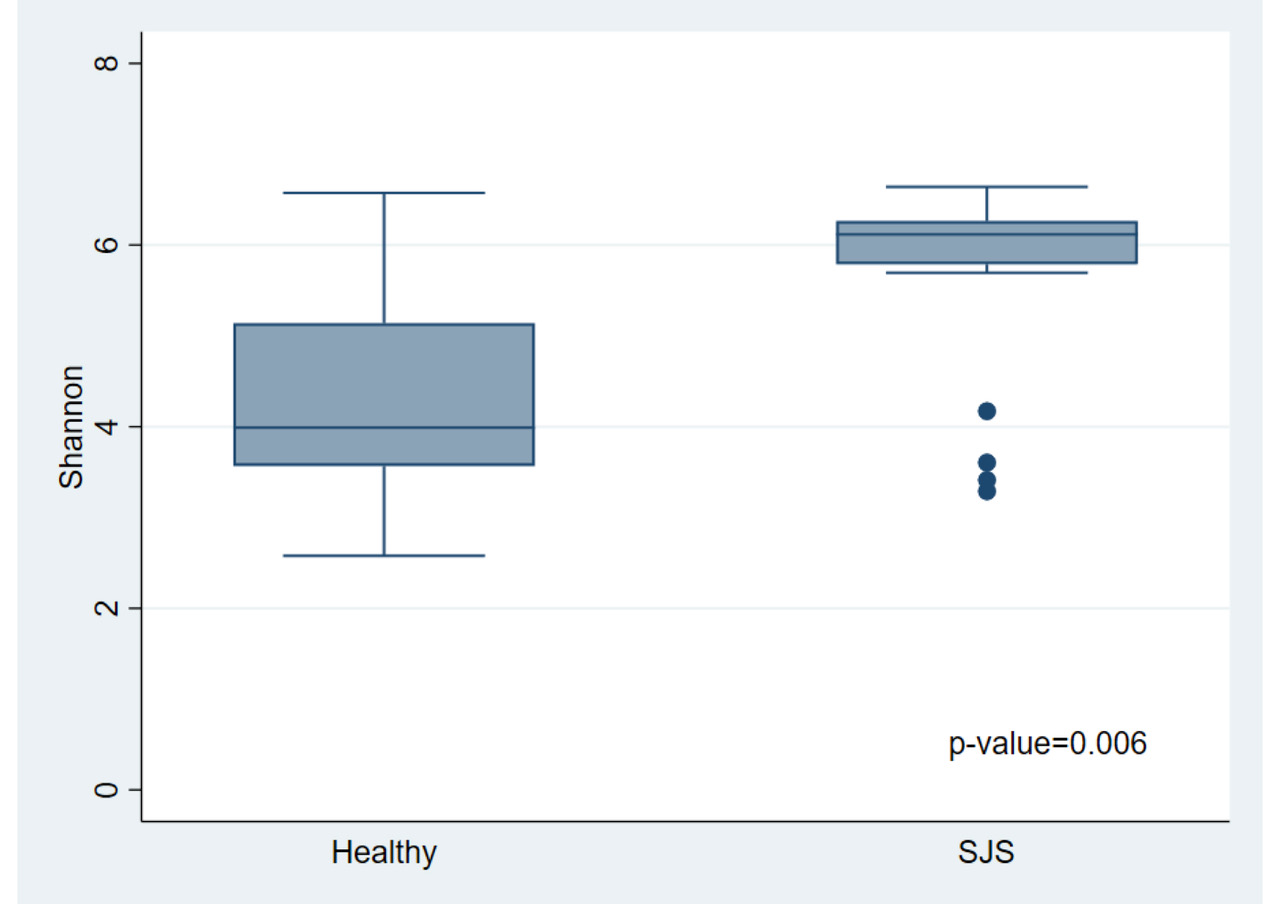

Supplement: Supplementary file 2 [file Image_2.JPEG]

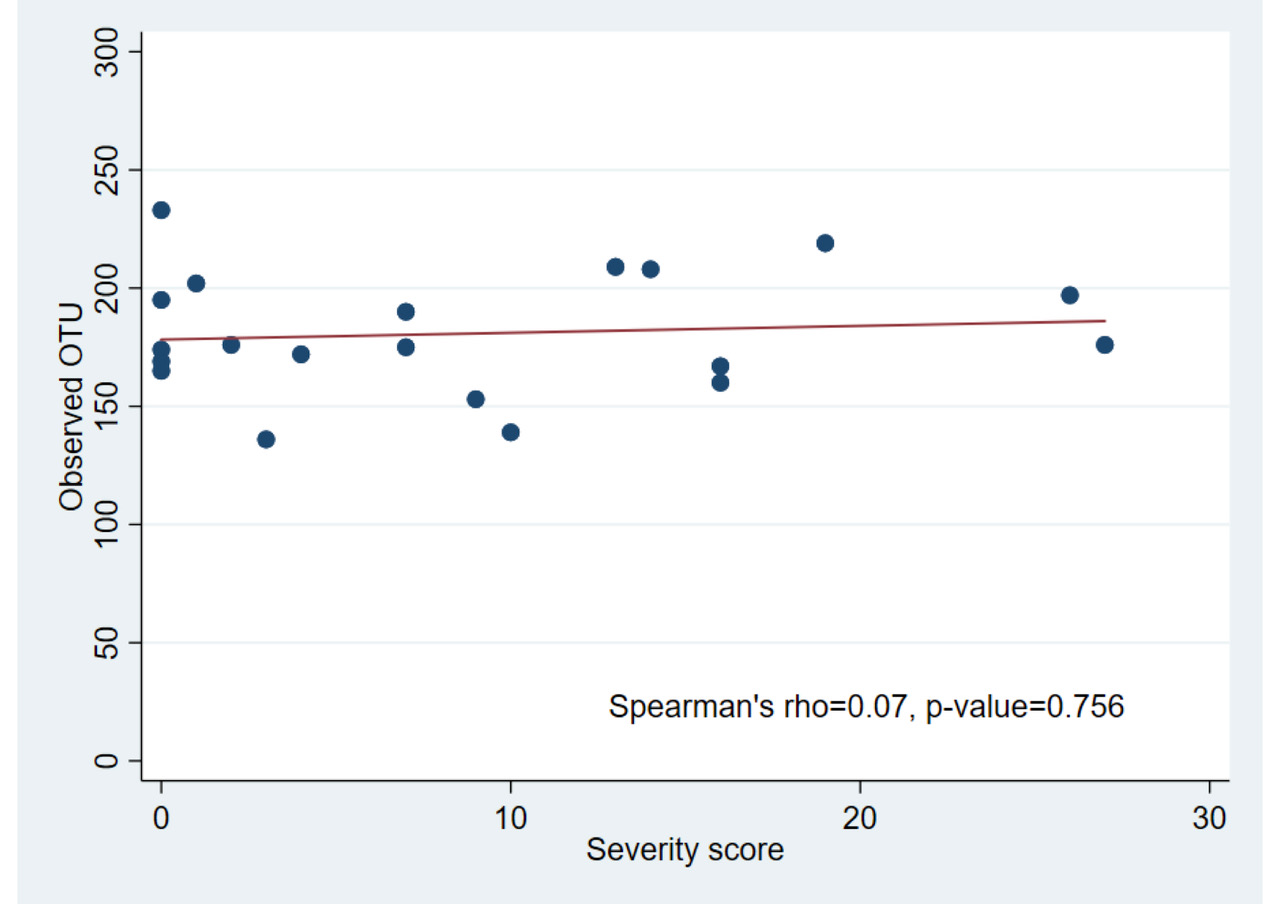

Supplement: Supplementary file 3 [file Image_3.JPEG]
